# Inactivation of *Pkd2* in adult mice results in delayed cyst formation and identifies sex as a major modifier of disease severity

**DOI:** 10.1016/j.kint.2025.08.039

**Published:** 2025-10-10

**Authors:** Patricia Outeda, Perry Summers, Denis Basquin, Ruizhen Xu, Jessica Hunt, Luis F. Menezes, Terry Watnick

**Affiliations:** 1 Division of Nephrology, Department of Medicine, University of Maryland School of Medicine, Baltimore, Maryland, USA; 2 Polycystic Kidney Disease Section, Kidney Disease Branch, National Institute of Diabetes and Digestive and Kidney Disease, National Institutes of Health, Bethesda, Maryland, USA

**Keywords:** ADPKD, animal model, gene expression

## Abstract

**Introduction:**

Autosomal Dominant Polycystic Kidney Disease (ADPKD) is caused by mutations in *PKD1* or *PKD2* and is the most common single-gene disorder resulting in kidney failure. Since deletion of either *Pkd1* or *Pkd2* in mice is embryonically lethal, conditional alleles have become indispensable tools for studying the pathobiology of ADPKD and for preclinical testing. The rapidity of cyst formation in P*kd1* conditional mice depends on the timing of gene inactivation, but this has never been systematically studied for *Pkd2*. Here, we characterize the kinetics of cystogenesis in a *Pkd2* conditional mouse model and identify associated alterations in gene expression.

**Methods:**

Using a conditional mouse model, we inactivated *Pkd2* at different postnatal stages and characterized the severity of cyst formation. Additionally, we performed RNA-sequencing analysis to identify gene expression changes associated with *Pkd2* loss in males and females.

**Results:**

There is a similar developmental switch in *Pkd2* conditional mice with delayed cyst formation after *Pkd2* inactivation at or beyond postnatal day 14. Like *Pkd1* models, cystogenesis is associated with differential expression of genes involved in metabolism, cell proliferation, and immune response. We confirm that sex is a key modifier of ADPKD progression with differences in disease severity occurring in the context of significant transcriptional differences between males and females that are independent of the *Pkd2* genotype.

**Conclusions:**

*Pkd2* conditional mice are a useful model for understanding ADPKD pathogenesis and for testing therapeutics. Our data highlight the importance of sex as a biological variable that should be considered in any study design using orthologous mouse models with late *Pkd*2 inactivation.

Autosomal dominant polycystic kidney disease (ADPKD) is a common genetic disorder that results in end-stage kidney disease because of the replacement of renal tissue with fluid-filled cysts.^1^ ADPKD is caused by mutations in *PKD1* or *PKD2*, encoding polycystin-1 and polycystin-2, respectively.^2-4^ The functions of polycystin-1 and polycystin-2 are incompletely understood. One model posits that polycystin-1 and polycystin-2 interact to form a receptor-channel complex that traffics to the primary cilia of renal epithelial cells, where it plays a critical role in maintaining tubular architecture.^5-10^ ADPKD progression is variable, and several factors have been recognized to affect disease severity. Population studies demonstrate that individuals with *PKD1* mutations develop end-stage kidney disease nearly 20 years earlier than those with *PKD2*.^11,12^ In addition, among those with *PKD1* mutations, protein truncating variants are associated with severe forms of PKD, whereas missense alterations are milder.^13^ However, even within these groups, there is significant variability, suggesting that there are other factors that impact disease progression.^14-16^

Mouse models are indispensable for studying the pathobiology of cystic disease and for testing preclinical therapies. Inactivation of *Pkd1* in mice during embryonic development or within the first 12 days after birth results in rapid cyst formation (early-onset model).^17-19^ In contrast, inactivation of *Pkd1* at or beyond postnatal day (P) 14 results in delayed cyst development (late-onset model) with a lag phase of $4 months, at which point there is rapid cyst formation and expansion.^17-19^ Further characterization suggested that this shift in the tempo of cyst initiation takes place on a background of diminishing proliferation and altered gene expression, marking a normal developmental switch that occurs between P11/12 versus P14/15 in wild-type kidneys.^18,20^ Further studies in the adult-onset *Pkd1* model revealed that males had a more severe phenotype compared with females and that sex was correlated with differential expression of genes involved in metabolic pathways and lipid metabolism.^21^

Although it is presumed that these findings can be extrapolated to *Pkd2* mutant mice, this has never been systematically tested.^22^ The objective of the present study was to characterize the kinetics of cystogenesis and disease progression in a *Pkd2* conditional mouse model and to identify associated alterations in gene expression.

## METHODS

### Animal care and use

All animal experimental procedures were conducted in accordance with the University of Maryland Animal Care and Use Committee guidelines and procedures. Animal models are described in the Supplementary Methods.

### RNA-sequencing analysis

RNA sequencing (RNA-seq) was performed at the Institute for Genome Sciences at the University of Maryland School of Medicine. Sequence files were preprocessed and trimmed using fastp 0.23.2^23^ and aligned to mm10 mouse genome using STAR 2.7.10b.^24^ Count matrices were imported into R 4.3.0^25^ and Rstudio 2023.03.0.386^26^ and analyzed with DESeq2^27^ with design = ~ group + comparison, where “group” controlled for litter/sex and comparison was either genotype or sex, depending on the analysis. Genes with Benjamini-Hochberg–adjusted *P* < 0.01 in the corresponding comparisons were considered differentially expressed. Pathway and transcription factor enrichment analyses were performed with clusterProfiler^28^ and databases in ChEA3.^29^ Protein-protein physical interaction was performed using STRINGdb^30^ and STRING mouse database version 12.0, with score threshold of 750 and “physical” network type. Plots were generated using ggplot2,^31^ ggvenn, UpSetR, enrichplot, and igraph packages in R.

Detailed methods can be found in the Supplementary Methods.

## RESULTS

### Timing of *Pkd2* inactivation determines the rate of PKD progression in mice

**Figure 1 ∣:**
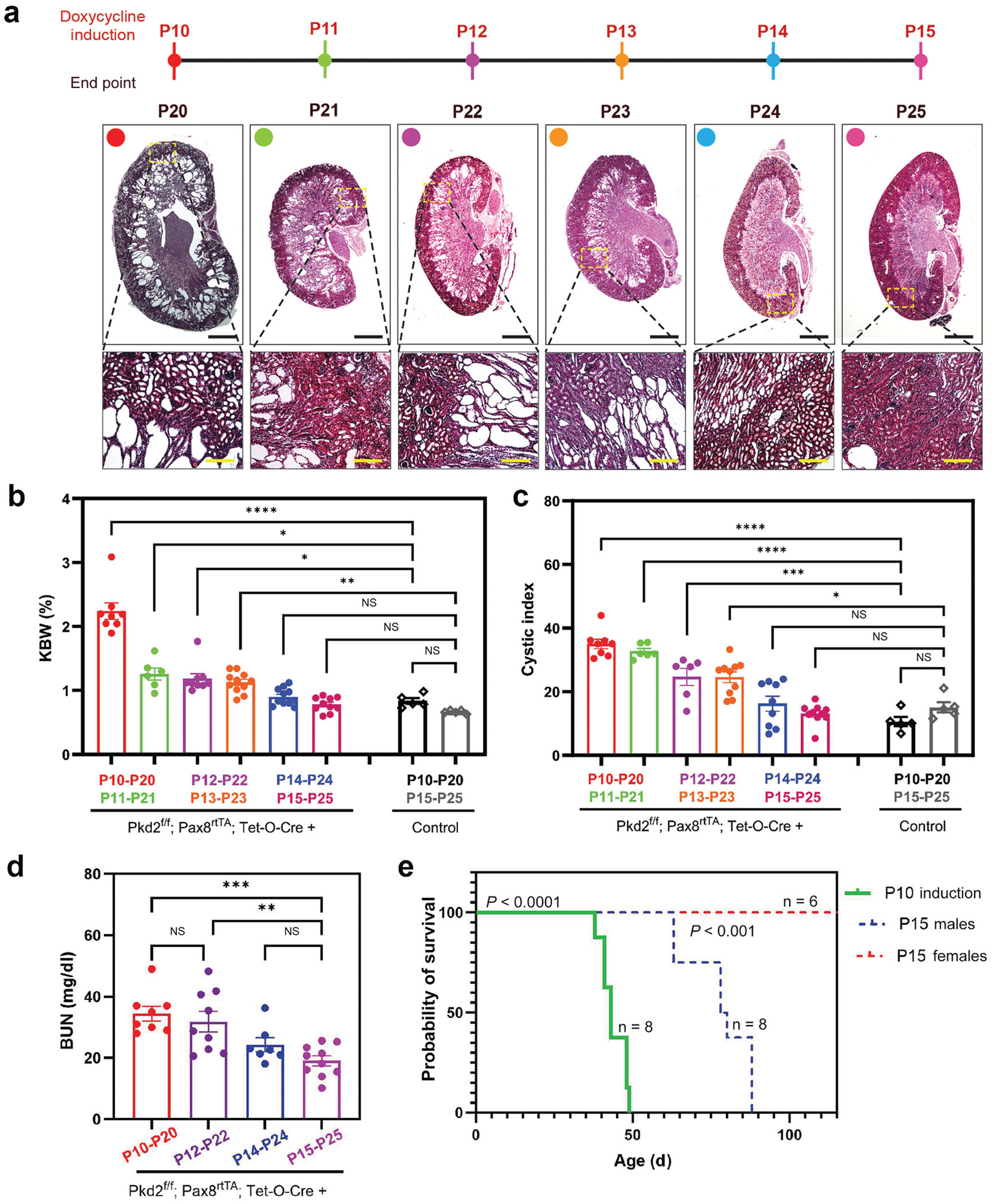
Timing of *Pkd2* inactivation in mice determines the rate of polycystic kidney disease (PKD) progression. (**a**) Representative histology of hematoxylin and eosin–stained kidney sections from *Pkd2*^*fl/fl*^; *Pax8*^*rtTA*^; *TetO*^*Cre*^ mice treated with a single i.p. dose of doxycycline (50 mg/kg) at postnatal day (P) 10 (N = 8), P11 (N = 6), P12 (N = 9), P13 (N = 11), P14 (N = 11), and P15 (N = 10) and harvested 10 days after *Pkd2* inactivation. Control mice lacked either the *Pax8*^*rtTA*^ or the *TetO*^*Cre*^ module and were treated with doxycycline at P10 or P15. Black bar = 2 mm; yellow bar = 250 μm. (**b**) Comparison of 1 kidney weight–to–body weight ratio (KBW). (**c**) Comparison of cystic index. (**d**) Comparison of blood urea nitrogen (BUN) levels. Overall, the phenotype was more variable with doxycycline induction at P13 and P14, which may be due to slight differences in age or stage of renal maturation. Colored bars represent timing of inactivation (red, P10; green, P11; purple, P12; orange, P13; blue, P14; and pink, P15). Solid circles indicate mutants; and diamonds, controls. Statistical significance was determined using multiple group comparisons by ordinary 1-way analysis of variance, followed by Tukey multiple comparisons test. Data are presented as mean ± SEM. *P < 0.05, **P < 0.01, ***P < 0.001, ****P < 0.0001. (**e**) Kaplan-Meier survival curves for mice induced at P10 (N = 8) (solid green line) compared with males (dashed blue line) and females (red dashed line) induced at P15 (N = 8 and N = 6, respectively), *P* < 0.0001. Statistical significance was calculated using the log-rank (Mantel-Cox) test to compare survival between the 3 groups. *P* < 0.001. Statistical significance between P15 males and P15 female survival curves was calculated using a pairwise log-rank (Mantel-Cox) test. NS, not significant (*P* > 0.05). To optimize viewing of this image, please see the online version of this article at www.kidney-international.org.

To characterize the kinetics of cyst formation, we treated *Pkd2^fl/fl^; Pax8^rtTA^; TetO^Cre^* mice with doxycycline (DOX), resulting in activation of Cre recombinase and *Pkd2* deletion throughout the nephron, including proximal and distal tubules and collecting ducts.^32,33^ To begin, we treated *Pkd2^fl/fl^; Pax8^rtTA^; TetO^Cre^* pups with a single i.p. injection of DOX at time points between P10 and P15. Kidneys were harvested 10 days after inactivation. Control mice (*Pkd2^fl/fl^; Pax8^rtTA^* or *Pkd2^fl/fl^; TetO^Cre^*) were treated with DOX and harvested at P20 or P25. As shown in Figure 1, kidneys from pups induced between P10 and P13 were grossly enlarged after 10 days, whereas kidneys induced at P14 and P15 had nearly absent cystic disease (Figure 1a and Supplementary Figure S1A). We quantified the kidney weight–to–body weight ratio (KBW) and found that pups induced at P10 to P13 (and harvested at P20 to P23) had a significant increase in KBW compared with controls. In contrast, the KBW of kidneys harvested at P24 to P25 after induction at P14 to P15 did not differ significantly from control kidneys harvested at P25 (Figure 1b). Hematoxylin and eosin staining confirmed an increased cystic index in pups induced at P10 to P13 (Figure 1c), whereas P24 and P25 kidneys from pups induced at P14 and P15, respectively, had no change in cystic index compared with controls (Figure 1c). Blood urea nitrogen (BUN) levels remained within the normal range in P24 and P25 pups that were induced at P14 and P15. In comparison, BUN levels at P20 and P22 in mice induced at P10 and P12 were significantly elevated (Figure 1d). The median survival for mice that were induced at P10 was 43 days, compared with a median survival of 79 days for males induced at P15 (*P* < 0.0001) (Figure 1e). All female mice were alive at P116. To determine whether differences in cyst progression were due to Cre-recombinase activity, we evaluated the efficiency of *Pkd2* deletion. We induced Cre recombination in pups at P10 and P15 and harvested kidneys 3 days later—at P13 and P18, respectively—before cyst formation began. We isolated renal tubules to enrich for epithelial cells, which are the primary targets of Cre-mediated recombination. Quantitative polymerase chain reaction analysis from renal tubules confirmed efficient deletion of *Pkd2* at both time points, with recombination levels of ≈82% to 85% (Supplementary Figure S1B and C).

### Inactivation of *Pkd2* at P15 results in delayed cyst formation with sexual dimorphism

**Figure 2 ∣:**
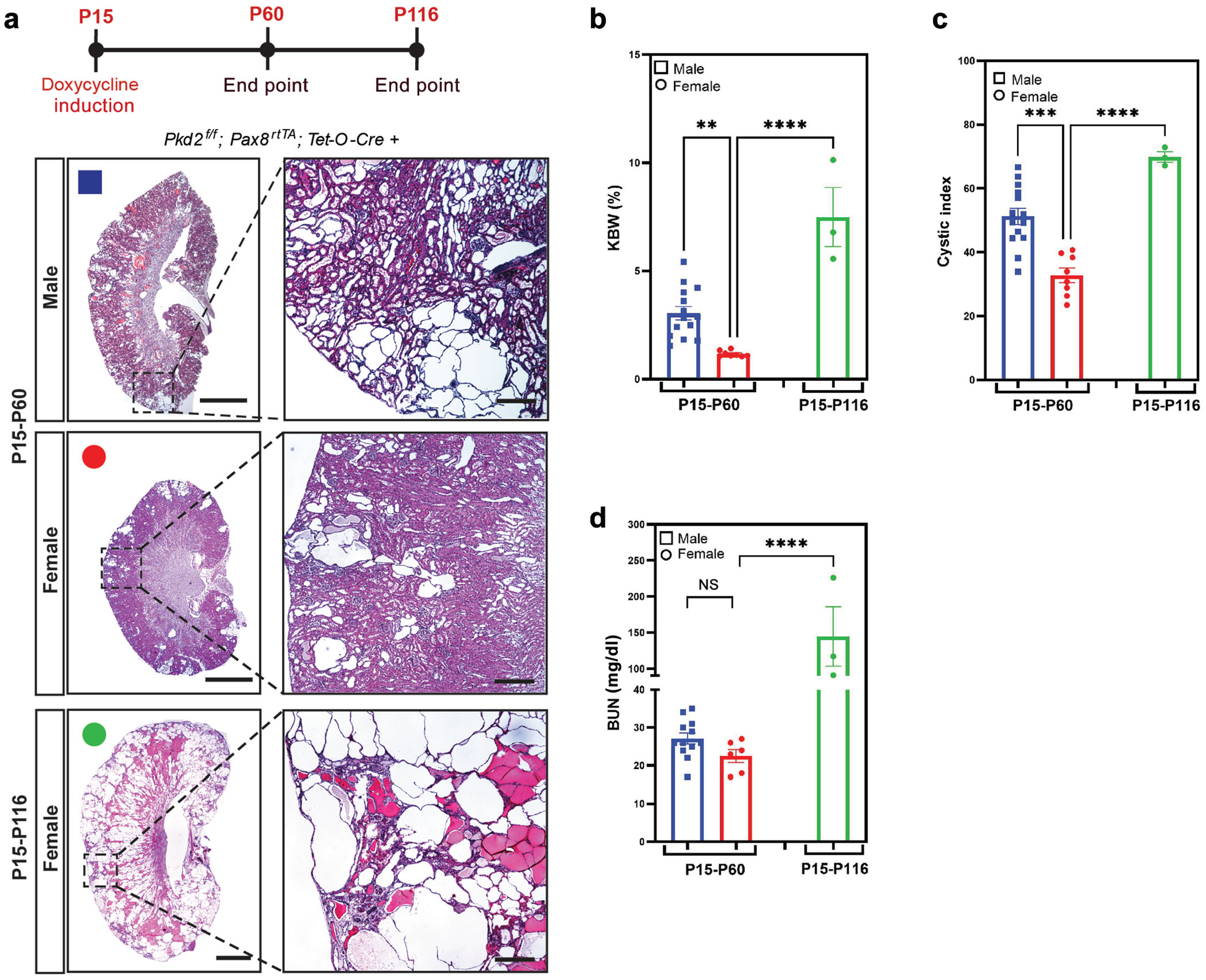
Inactivation of *Pkd2* at postnatal day 15 (P15) results in delayed cyst formation with sexual dimorphism. (**a**) Representative histology of hematoxylin and eosin–stained kidney sections from *Pkd2*^*fl/fl*^; *Pax8*^*rtTA*^;*TetO*^*Cre*^ mice induced with a single i.p. dose of doxycycline (50 mg/kg) at P15 and harvested at P60, 45 days after *Pkd2* inactivation, from males (N = 14) (blue squares) and females (N = 8) (red circles) and from females harvested at P116, 101 days after *Pkd2* inactivation (N = 3) (green circles). Bar = 2 mm (left) and 200 μm (right). Quantitative data for (**b**) 1 kidney weight–to–body weight ratio (KBW), (**c**) cystic index, and (**d**) blood urea nitrogen (BUN) levels from males (N = 12) (blue squares), females (N = 6) (red circles), and from females harvested at P116 (N = 3) (green circles). Statistical significance was determined using multiple group comparisons by ordinary 1-way analysis of variance followed by Tukey multiple comparisons test. Data are presented as mean ± SEM. **P < 0.01, ***P < 0.001, ****P < 0.0001. NS, not significant (*P* > 0.05). To optimize viewing of this image, please see the online version of this article at www.kidney-international.org.

To determine the time course of cyst development after induction at P15, we collected kidneys at P60 (45 days after *Pkd2* inactivation). By this time, all mice had evidence of cyst formation (Figure 2a and Supplementary Figure S2A). However, at P60, the KBW and cystic index were significantly reduced in females versus males (Figure 2b and c and Supplementary Figure S2A). Although BUN levels were slightly higher in males compared with females, the difference was not statistically significant (Figure 2d). Analysis of the kidney phenotype in females induced at P15 and harvested at P116 confirmed progression to a severe phenotype, with elevated KBW ratios, cystic index, and BUN levels (Figure 2a-d and Supplementary Figure S2B). Taken together, these findings suggest delayed disease progression in females versus males, with cyst burden ultimately reaching a similar degree of severity. In contrast, we did not observe any difference in KBW or cystic index between males and females in mice induced before P14 or in mice induced at P15 but harvested before the onset of cystogenesis (Supplementary Figure S3).

To exclude the possibility of sex differences in Cre activity as a cause of sexual dimorphism in disease progression, we generated *Pkd2^fl/fl^; Pax8^rtTA^; TetO^Cre^* mice with the double-fluorescent *mT/mG* reporter (*Pkd2^fl/fl^; Pax8^rtTA^; TetO^Cre^*; *mT/mG*).^34^ Following induction at P15 and analysis at P20, we observed comparable green fluorescent protein expression in the cortex and the medulla between sexes, supporting the idea that differences in cyst formation are not due to Cre recombinase activity (Supplementary Figure S4A). Additionally, we performed Western blot analysis on kidneys from pups induced at P15 and collected at P25, before cyst formation and observed a ~75% reduction in polycystin-2 expression in both sexes compared with controls (Supplementary Figure S4B and C).

We stained P60 kidney sections with Masson trichrome and anti–α-smooth muscle actin, both markers of fibrosis. Although there was no difference in collagen deposition between males and females, there was an increase in myofibroblasts around developing cysts that was more pronounced in males (Supplementary Figure S5).

### Kidney cysts develop asynchronously after *Pkd2* inactivation

**Figure 3 ∣:**
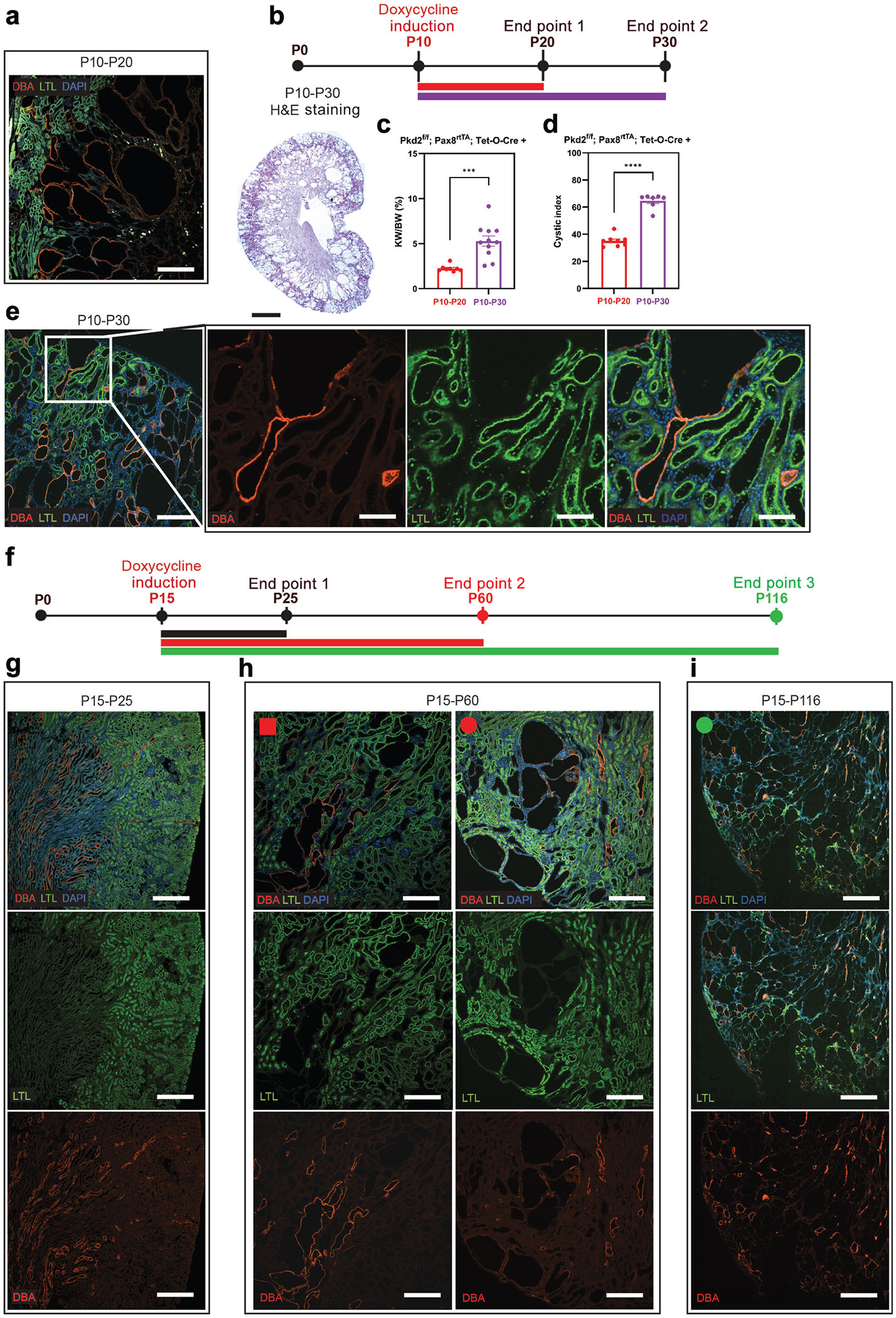
Kidney cysts develop asynchronously after *Pkd2* inactivation. (**a**) Representative kidney image from a *Pkd2*^*fl/fl*^; *Pax8*^*rtTA*^; *TetO*^*Cre*^ mouse induced at postnatal day 10 (P10) with i.p. doxycycline (50 mg/kg) and harvested at P20 (N = 8). Sections were stained with markers for proximal tubules (*Lotus lectin* [LTL]; green), distal tubules (*Dolichos biflorus agglutinin* [DBA]; red), and nuclei (4′ ,6-diamidino-2-phenylindole [DAPI]; blue) and show the presence of large distal cysts and modestly dilated proximal tubules. (**b**) Representative histology of a hematoxylin and eosin (H&E)–stained kidney section from a mouse treated with doxycycline at P10 as in (**a**) and harvested 20 days after *Pkd2* inactivation at P30 (N = 11). (**c,d**) Comparison of (**c**) 1 kidney weight (KW)/body weight (BW) ratio or (**d**) cystic index for kidneys treated with i.p. doxycycline at P10 and harvested at P20 (red circles; N = 8) versus P30 (purple circles; N = 11). Statistical significance was determined using unpaired 2-tailed *t*-test. Data are presented as mean ± SEM. ***P < 0.001 and ****P < 0.0001. (**e**) Representative kidney section from mouse induced at P10 (as in [**a**]) and harvested at P30, stained with markers for proximal tubules (LTL; green), distal tubules (DBA; red), and nuclei (DAPI; blue). (**f**) Schematic timeline showing experimental events. Mice were induced with doxycycline (50 mg/kg; i.p.) at P15. Kidneys were harvested at P25 (black dot), P60 (red dot), and P116 (green dot). (**g**) Representative kidney section collected from a mouse induced with doxycycline at P15 and harvested at P25 and stained with markers for proximal tubules (LTL; green), distal tubules (DBA; red), and nuclei (DAPI; blue) showing the absence of cysts. (**h**) Representative kidney sections collected from a male (red square; left panel) and a female (red circle; right panel) mouse induced with doxycycline at P15 and harvested at P60. (**i**) Representative kidney section collected from a female (green dot) induced with doxycycline at P15 and harvested at P116. Sections were stained with markers for proximal tubules (LTL; green) and distal tubules (DBA; red). In the male, cysts stained with LTL and DBA, indicating both proximal and distal tubule origin. Females (right panel; red circle) had distal cysts with only modestly dilated proximal tubule. Larger cysts in both sexes often lacked staining for either LTL or DBA. Females at P116 (green dot) exhibited a more severe phenotype compared with earlier time points (P60), indicating a delayed disease progression. Bars = 2 mm (black); 250 μm (white). To optimize viewing of this image, please see the online version of this article at www.kidney-international.org.

To determine the origin of cysts after *Pkd2* inactivation, we performed immunofluorescence staining of kidney sections with *Lotus lectin* (LTL) and *Dolichos biflorus agglutinin* (DBA), marking proximal and distal tubules, respectively. We found that kidney sections collected at P20 after induction at P10 exhibited large DBA-positive cysts, whereas LTL-positive proximal tubules were discreetly dilated or not dilated (Figure 3a). Examination of renal sections from kidneys harvested at P30 (after *Pkd2* inactivation at P10) showed that cystic disease had progressed, as evidenced by an increase in KBW and cystic index between P20 and P30 (Figure 3b-d). By this time, cysts clearly displayed proximal and distal tubule markers, with areas of some larger cysts appearing to lose segment-specific markers (Figure 3e).

Next, we investigated cyst origin in *Pkd2^fl/fl^; Pax8^rtTA^; TetO^Cre^* mice induced at P15 (Figure 3f). As expected, there were no kidney cysts at P25 (Figure 3g). By P60, however, we observed cysts arising from both proximal and distal tubules in males (Figure 3h, left panel), whereas in females, cysts arose mostly from distal tubules (Figure 3h, right panel). By P116, females exhibited cysts staining for both proximal and distal tubule markers, similar to males at P60 (Figure 3i). To determine whether there were regional differences in Cre activity, we induced *Pkd2* deletion in *Pkd2^fl/fl^; Pax8^rtTA^; TetO^Cre^*; *mT/mG* mice at P10 or P15 and collected tissues at P15 or P20, respectively. We observed comparable green fluorescent protein expression in both the medulla and the cortex, suggesting robust Cre activity in both proximal and distal tubules (Supplementary Figure S4A and Supplementary Figure S6).

To explore the role of proliferation in cyst formation, we costained kidney sections from *Pkd2^fl/fl^; Pax8^rtTA^; TetO^Cre^* mice induced at P15 and harvested at P60 with Ki67, along with LTL and DBA (Supplementary Figure S7). We observed a significantly higher proportion of proliferative (Ki-67^+^) cells in DBA^+^ collecting duct segments from males (~25%) compared with females (~15%). In proximal tubules (LTL^+^ segments), there was a trend toward increased proliferation in males (Supplementary Figure S7). These findings suggest that enhanced proliferation may contribute to the severe cystic phenotype observed in males.

### Transcriptomic analysis of *Pkd2* mutant kidneys reveals sex as a major determinant of gene expression

We used RNA-seq to investigate the transcriptional underpinnings of cyst formation in *Pkd2* mutant kidneys. We decided to focus on the late induction model because this would also allow us to correlate the observed phenotypic differences between males and females with gene expression profiles. We induced *Pkd2* deletion in *Pkd2^fl/fl^; Pax8^rtTA^; TetO^Cre^* mice with i.p. DOX at P27 to P29 to avoid the critical window around P14 that determines the kinetics of cyst formation. In addition, this strategy minimizes small differences in the timing of parturition or gestational age. We harvested kidneys at P85 and P125 from 10 males and 10 females for each time point. For each sex, there were N = 5 Cre-positive and N = 5 Cre-negative controls.

**Figure 4 ∣:**
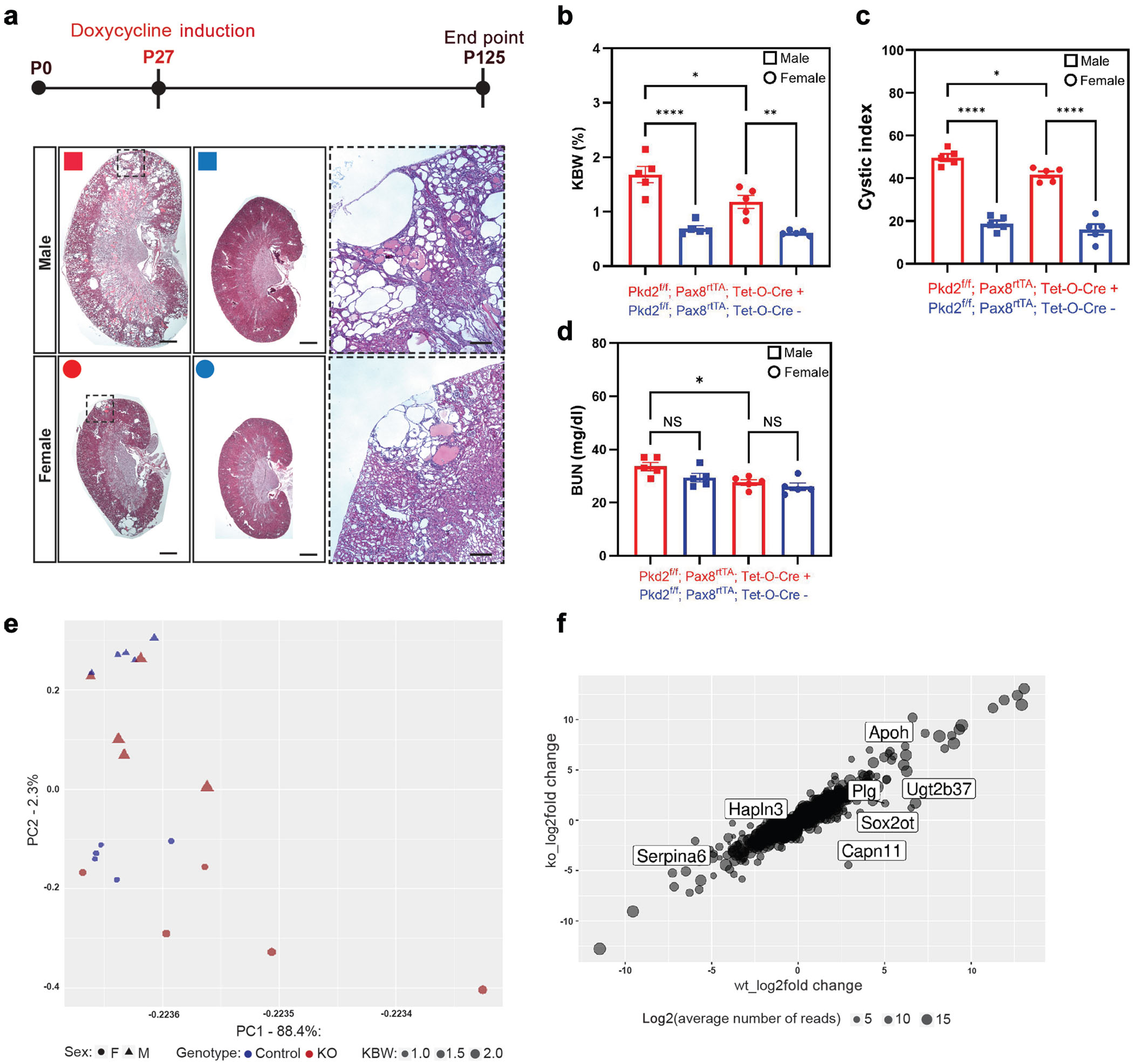
Transcriptomic analysis of *Pkd2* mutant kidneys reveals sex as a major determinant of gene expression. (**a**) Representative hematoxylin and eosin–stained kidney sections from *Pkd2*^*fl/fl*^; *Pax8*^*rtTA*^; *TetO*^*Cre*^ male (M) and female (F) mice and control littermates treated with i.p. doxycycline (50 mg/kg) at postnatal day (P) 27 to P29 and harvested at P125. Bars = 2 mm and 200 μm. Comparison of (**b**) 1 kidney weight–to–body weight ratio (KBW), (**c**) cystic index, and (**d**) blood urea nitrogen (BUN) levels from P125 mutant (red) and control (blue) males (squares) and female (circles) kidneys (N = 5/group/sex) used for transcriptomic analysis. Data are presented as mean ± SEM. Statistical significance was determined using multiple group comparisons by ordinary 1-way analysis of variance followed by Tukey multiple comparisons test. *P < 0.05, **P < 0.01, and ****P < 0.0001. (**e**) Principal component (PC) plot representing global transcriptional similarities between samples. Note that the second PC mostly separates sex (triangles: males; circles: females). Within sexes, mutants (red) and controls (blue) are also clustered. Circle and triangle sizes represent KBW. (**f**) Plot showing log2 fold change of male to female comparisons in either wild-type controls (wt.; *x* axis) or mutants (knockout [ko]; *y* axis) for all the genes in the panel. Genes where the fold-change difference between mutant and control groups was >3 (off the diagonal) are labeled. Circle size correlates with the number of reads. NS, not significant (*P* > 0.05). To optimize viewing of this image, please see the online version of this article at www.kidney-international.org.

At P85, both males and females had early-stage PKD, but cyst formation was more advanced in males, with a statistically significant increase in KBW and cystic index compared with same-sex controls (Supplementary Figures S8 and S9). Females had scattered cysts with a minimal increase in cystic index and no change in KBW. By P125, both male and female kidneys showed more advanced cystic disease with KBW, and cystic index significantly increased compared with controls of the same sex (Figure 4a-c and Supplementary Figure S8). Consistent with observations at P60 and P85, males at P125 continued to have more pronounced cystic disease with higher BUNs compared with females (Figure 4d).

We conducted bulk RNA-seq analysis of the kidneys harvested at P125. In Figure 4e, the principal component analysis, representing transcriptional similarities between samples, shows that sex is a more significant determinant of sample clustering than *Pkd2* mutation status. To determine whether sex-dependent gene expression differences were genotype independent, we plotted log2 fold change of male-to-female comparisons for controls (*x* axis) and mutants (*y* axis). We found that the fold-change alterations of differentially expressed genes (DEGs) between sexes in both control and mutant groups show a strong correlation (Pearson correlation = 0.94), suggesting that sexual dimorphism in gene expression is independent of genotype (Figure 4f and Supplementary Figure S10A). Transcription factor enrichment analyses linked estrogen receptor α (ESR1), a hormone receptor that mediates the pleiotropic effects of steroid hormones in males and females, with DEGs in both controls and mutants (Supplementary Figure S10B and C).^35-37^ Western blot analysis confirmed that the expression level of ESR1 was higher in both male and female mutant P125 kidneys compared with controls (Supplementary Figure S10D and E).

**Figure 5 ∣:**
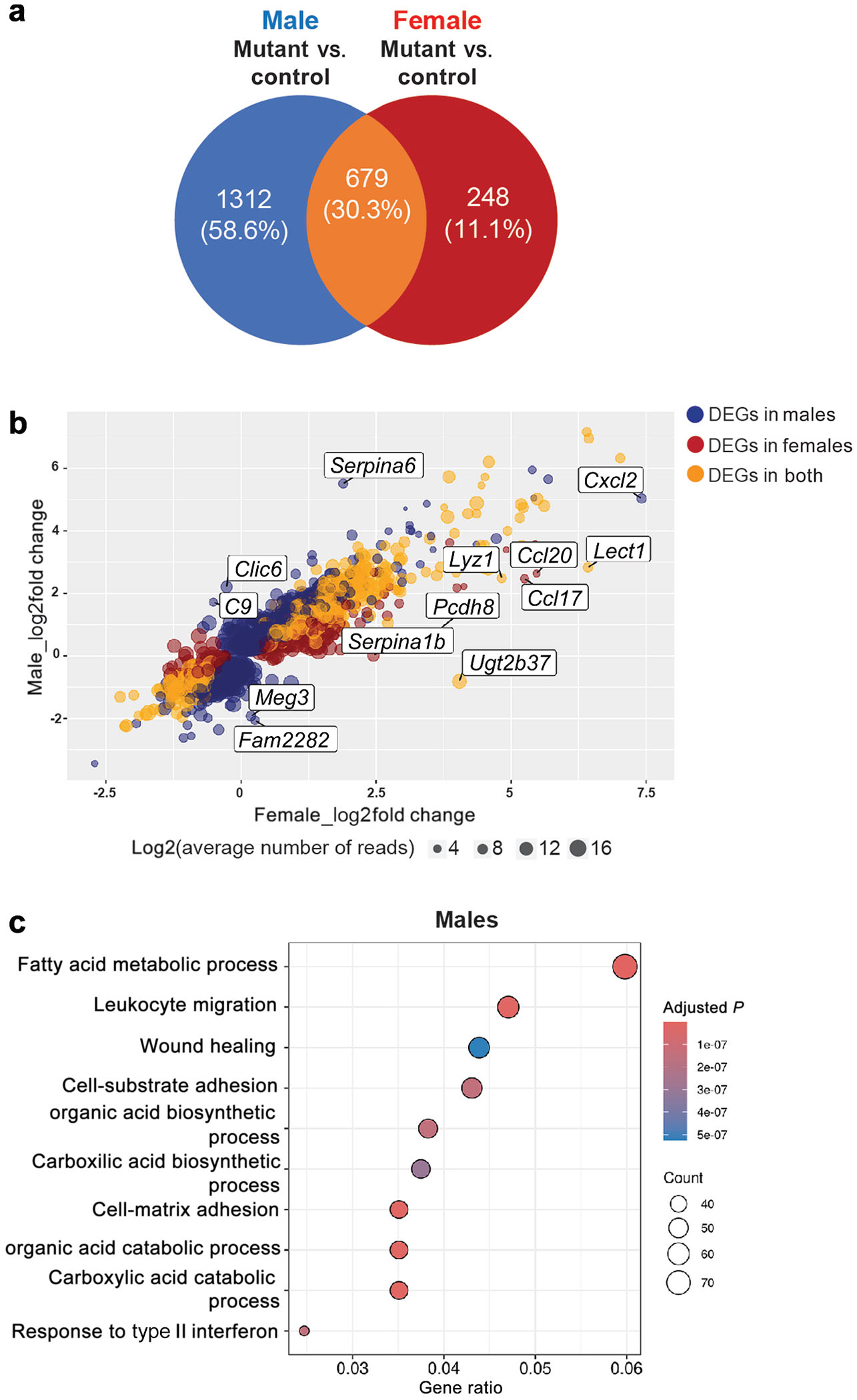
Genotype-specific transcriptional differences by gender. (**a**) Venn diagram showing differentially expressed genes (DEGs) (adjusted *P* < 0.01) comparing mutants with controls in males (blue), females (red), and both orange). (**b**) Plot showing log2 fold change of mutant to control comparisons in either females (*x* axis) or males (*y* axis) for genes in panel (**a**). Genes where the fold-change difference between mutant and control groups was >3 (off the diagonal) are labeled. Circle size correlates with the number of reads. Genes differentially expressed only in males are colored in blue; in females, colored in red; and in both sexes, colored in orange. (**c**) Dot plot showing top enriched biological process pathways in genes that were differentially expressed only in males.

Because males tend to have more severe PKD in our mouse model and in previously published *Pkd1* models,^21,38^ we hypothesized that male-specific, genotype-dependent differences in gene expression might provide clues about disease-modifying pathways. To address this, we compared transcriptional differences between mutants and controls by sex and identified genotype-specific transcriptional differences unique to males or females (Figure 5a and b). As shown in Figure 5a, ~59% of the DEGs were specific to males, whereas ~11% were specific to females, and only ~30% of the DEGs were shared. Despite this, we found a high overall correlation in fold-change expression between mutants versus controls when compared by sex (Pearson correlation = 0.91) (Figure 5b). In fact, even genes statistically significant in only 1 sex showed correlated fold changes between males and females (Pearson correlation = 0.86), suggesting shared cystogenic pathways in males and females. Pathway analysis of DEGs specific to mutant versus control males demonstrated that the most highly enriched biological processes were associated with metabolic pathways (including fatty acid and carboxylic acid metabolism), chemotaxis, and cell migration (Figure 5c). We conducted a similar analysis in females but failed to identify pathways with comparably low *P* values and gene enrichment, likely because the number of DEGs unique to females was too small for reliable pathway identification.

### Gene expression patterns in *Pkd2* mutant kidneys correlate with KBW

**Figure 6 ∣:**
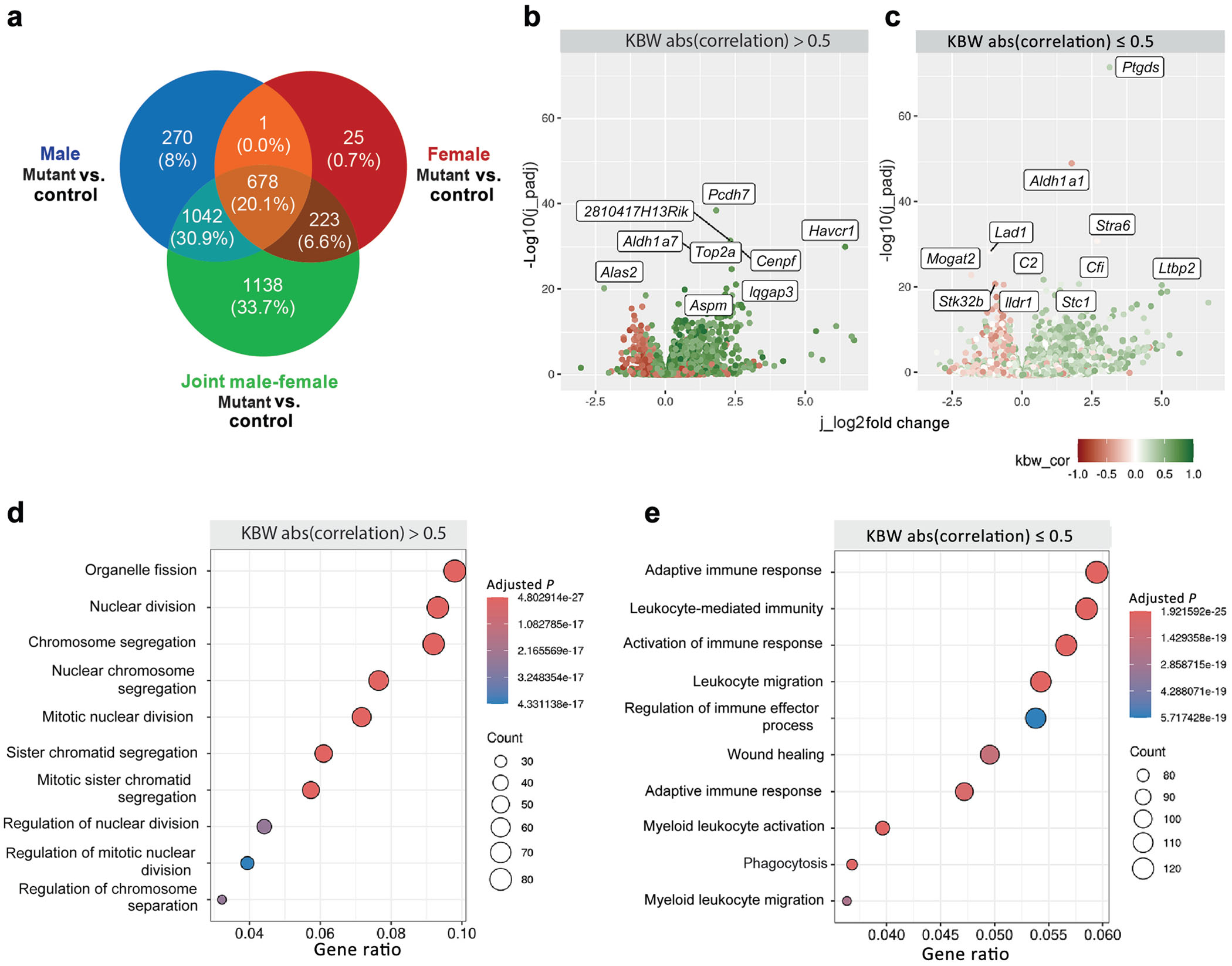
Joint analysis of transcriptional differences in *Pkd2* mutant kidneys and correlation with kidney weight–to–body weight ratio (KBW). (**a**) Venn diagram of differentially expressed genes (adjusted *P* < 0.01) in mutants versus controls only in males, only in females, or in a combined male and female analysis controlled by sex. (**b,c**) Volcano plots with genes differentially expressed in the combined male and female analysis (joint male-female), colored on the basis of their absolute correlation with KBW (high correlation: dark red and dark green; low correlation: light red and light green). (**b**) Genes with an absolute (abs) correlation to KBW of >0.5. (**c**) Genes with an absolute correlation to KBW of ≤0.5. Genes with adjusted *P* < 10^−20^ are labeled. (**d,e**) Dot plot showing ratio of genes in the differentially expressed group to genes in the category (*x* axis) for enriched biological process pathways in genes that were differentially expressed and had (**e**) low (≤0.5) or (**d**) high (>0.5) absolute correlation with KBW.

Our analysis of differential gene expression in male and female mutants and controls suggested that the transcriptional landscape underlying cyst formation is similar in both sexes albeit delayed in females. In this scenario, there could be a core set of DEGs associated with cystogenesis that might have been missed in sex-stratified analyses but reach significance in a combined dataset. To test this, we compared all *Pkd2* mutant kidneys with controls, controlling for litter and sex. This analysis identified 3081 DEGs (adjusted *P* < 0.01, controlled by litter and sex), exceeding the number found in same-sex comparisons (Figure 6a). The observation that some genes reached statistical significance only in the combined male/female analysis suggests that certain gene expression changes are subtle but consistent across sexes.

To ascertain DEGs that might be relevant for cyst formation or cyst growth, we correlated DEGs with KBW in mutant samples (Figure 6b and c). Pathway analysis was conducted separately for those genes that had a high absolute correlation with KBW (high, >0.5 or <−0.05) (Figure 6b and d) or had little absolute correlation with disease progression as measured by KBW (low, <0.05 or >−0.05) (Figure 6c and e). We found that genes that were highly correlated with KBW ratios (either >0.5 or <−0.05) were enriched in pathways related to organelle fission and cell proliferation (Figure 6d). Among these was cyclin-dependent kinase-1 (*Cdk1*), which was previously reported to be dysregulated in *Pkd1* ADPKD mouse models.^39^ Genes with relatively low or no correlation with KBW ratios were enriched in pathways involved in the immune response (Figure 6e).

### Meta-analysis of the *Pkd2* transcriptome

To date, several transcriptomic studies have reported changes in animal and cell models after *Pkd2* inactivation.^39-43^ We reasoned that a joint analysis of our dataset with previously published datasets might identify a core set of transcriptional changes related to *Pkd2* loss. Among publicly available RNA-seq datasets, we selected 2 studies for further analysis. The first study (denoted as Somlo) included 3 mutants and 3 control kidneys from a mouse line like ours, *Pkd2^fl/fl^; Pax8^rtTA^; TetO^Cre^* (GSE149739).^39^ To obtain *Pkd2* knockout, the mice were administered oral DOX from P28 to P42, and kidneys were harvested at P70. At this point, mice were reported to have mild tubule dilation but no overt cysts. Both males and females were combined in this dataset. The second study (denoted as Welling) used an inducible immortalized mouse inner medullary collecting duct cell line derived from *Pkd2^fl/fl^; Pax8^rtTA^; TetO^Cre^; SV40 LTA^ts^* mice (GSE171574; N = 8 mutants and N = 8 controls).^41^ The immortalized conditional cell lines were treated with DOX to induce *Pkd2* loss or were vehicle treated. These datasets were chosen because they showed a consistent reduction in *Pkd2* transcript in mutant samples , had at least 3 samples per genotype, and because <30% of the genes were differentially expressed.

**Figure 7 ∣:**
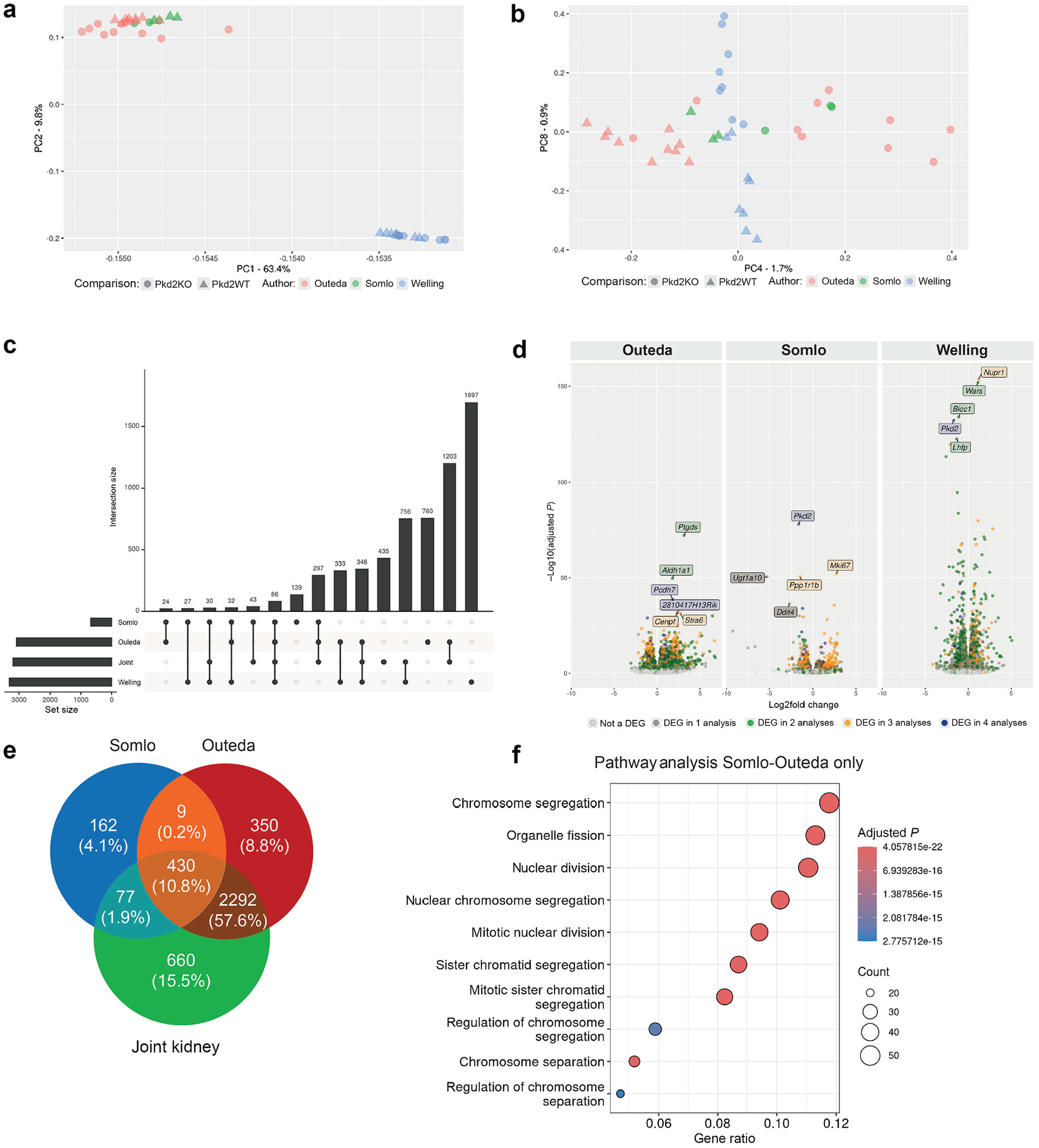
Meta-analysis of the *Pkd2* transcriptome. (**a,b**) Principal component (PC) plots representing transcriptional profile differences in 3 independent *Pkd2* knockout studies (circles: mutant samples; triangles: controls). Blue: inner medullary collecting duct (IMCD) cells (denoted as Welling)^41^; green: P70 kidneys (denoted as Somlo)^40^; red: P125 kidneys in the current study (denoted as Outeda). (**a**) Plot showing PC1 (*x* axis) and PC2 (*y* axis). (**b**) Plot showing PC4 (*x* axis) and PC8 (*y* axis). (**c**) Upset plot showing the size of overlap between the different analyses. A set of 86 genes was differentially expressed in all datasets and when the datasets were analyzed jointly (“joint all”). A set of 383 (297 + 86) were differentially expressed in the joint and kidney datasets (with or without differential expression in the IMCD set). The kidney datasets shared 439 differentially expressed genes (DEGs). (**d**) Volcano plots showing log2(fold change) of mutant to control (*x* axis) and corresponding –log10(adjusted *P* value) (*y* axis) of genes in each individual dataset. Dots representing genes are colored on the basis of the number of analyses in which they were found to be differentially expressed. Light gray: indicates genes that were not differentially expressed. Dark gray: indicates DEGs in 1 analysis. Green: indicates DEGs in 2 analyses. Orange: indicates DEGs in 3 analyses. Blue: indicates DEGs in 4 analyses (the joint analysis and each individual dataset). The top 5 DEGs in each dataset are labeled, and the boxes are colored on the basis of the number of analyses in which they were found to be differentially expressed (see above). (**e**) Venn diagram showing DEGs (with adjusted *P* < 0.01) in mutant versus controls in the kidney datasets (Somlo and Outeda) and in a joint analysis of the kidney datasets (joint kidney). (**f**) Pathway analysis of the 439 genes differentially expressed in both kidney datasets, showing dot plot of the ratio (*x* axis) of differentially expressed genes in the category to total number of genes in the category for each enriched biological process (*y* axis). Dots are colored on the basis of adjusted *P* value of pathway enrichment.

We reanalyzed the 2 previously published datasets for consistency and then conducted a meta-analysis. Principal component plots show that most of the transcriptional differences between samples can be accounted for by the tissue of origin (Figure 7a), and ≈2% of the variance can be explained by genotype (Figure 7b). The joint analysis of all 3 datasets (“joint all”) had a higher number of DEGs (N = 3196) than any individual dataset because statistical significance was achieved only when the data were combined. The upset plot (Figure 7c) and Venn diagram (Supplementary Figure S11A) show the DEGs that were unique to each dataset. There were only 86 DEGs that were shared across all the analyses (Figure 7c, Supplementary Figure S11A, and Supplementary Table S1). In Figure 7d, we color coded genes based on the number of analyses in which they were differentially expressed (each dataset separately and the joint all analysis). Some of the top DEGs in each individual dataset were not identified as differentially expressed in the “joint all” analysis, likely reflecting differences in background, tissue type, and/or disease state.

Because many of the differences in gene expression in the “joint all” analysis were driven by the large number DEGs in the cell line study, we performed a further evaluation of only the kidney datasets. Despite the difference in the number of DEGs (based on *P* value), the fold change in gene expression between mutants and controls in the 2 datasets was highly correlated (Pearson correlation = 0.95) (Supplementary Figure S11B). We then focused on a set of 439 genes that were differentially expressed in the kidney datasets (Figure 7e and Supplementary Table S2). Pathway analysis of the 439 DEGs in the kidney datasets identified enrichment for processes in cell division and proliferation (Figure 7f). Additional network analysis of 383 DEGs shared between the kidney datasets and the joint analysis showed associations with transcription factors Nanog homeobox (NANOG) and tumor protein P63 (TRP63), linked to proliferation and apoptosis pathways, respectively (Supplementary Figure S12A and B). STRING analysis of protein-protein interactions suggests that several DEGs in the joint all and kidney studies encode physically interacting proteins (Supplementary Figure S13), forming 2 subnetworks with 9 and 21 nodes (Supplementary Figure S14). These subnetworks are enriched in biological processes related to proliferation (Supplementary Figure S14A and B) and immune response (Supplementary Figure S14C and D).

## DISCUSSION

Several investigators have demonstrated that *Pkd1* inactivation in conditional mouse models before P14 leads to rapid cyst formation within weeks. In contrast, inactivation of *Pkd1* after weaning results in severe cystic kidney disease after a lag phase of several months.^17-19^ These findings have been consistent regardless of the Cre recombinase or *Pkd1* conditional mouse line. Careful mapping of this “breakpoint” identified a critical window between P12 and P14 that determines the disease kinetics.^18^ This switch corresponds to the end of kidney development and is characterized by reduced cell proliferation and by metabolic reprogramming, suggesting that the response to *Pkd1* mutation is determined by the renal context in which polycystin signaling is diminished.^18,20^

Here, we show for the first time that a similar time-dependent switch occurs with *Pkd2* deletion. Using a *Pkd2* conditional model combined with a DOX-inducible Cre driven by the *Pax8* promoter, we found that deletion of *Pkd2* at or before P12 triggers rapid cystogenesis, whereas deletion at or after P15 yields a delay of months before the onset of cystogenesis. This difference is unrelated to the degree of Cre recombinase activity in early versus late induction models. As has been described for *Pkd1*, we observed variability in the severity of cyst formation when *Pkd2* loss was induced at P13 to P14, likely because of subtle differences in age (timing of parturition) or in the stage of renal development.^18^ These findings differ from a previous study where embryonic *Pkd2* inactivation using *Aqp2*-Cre caused severe PKD, but postnatal inactivation at P1 produced no cysts.^22^ Because early postnatal inactivation of *Pkd1* in distal tubules is sufficient to drive cystogenesis, this discrepancy may reflect differences in Cre drivers and induction strategies.^17,22,44^ Further comparative studies are needed to determine how segment-specific inactivation affects cyst formation kinetics after *Pkd2* inactivation.

We also show that cysts in this *Pkd2* model arise from both proximal and distal tubules, consistent with Pax8-driven Cre activity. However, we found that cysts develop asynchronously despite little difference observed in Cre activity. We looked at the origin of cysts shortly after *Pkd2* deletion in both the early-onset and delayed-onset models and found that distal tubule (DBA-positive) cysts appear earlier than proximal tubule (LTL-positive) cysts, suggesting that the distal tubule/collecting duct may be more sensitive to the loss of polycystin signaling.

Another notable finding of our study is that although the severity of cystic disease was similar between males and females in the early-onset model, there was sexual dimorphism in the setting of late *Pkd2* inactivation. After *Pkd2* deletion at or beyond P15, males progressed faster with a higher KBW and cystic index in comparison with females. In contrast, females exhibit delayed cystogenesis and survive longer. This is similar to what has been described for *Pkd1* conditional models and for human ADPKD and is unrelated to Cre recombinase activity.^21,38,45^ One possible explanation for the lack of sexual dimorphism in the early-onset model is the absence of hormonal effects before the onset of sexual maturity, which occurs between 6 to 8 weeks of age in mice.^46,47^

In keeping with the importance of “renal context” as a factor in PKD progression, RNA-seq analysis of kidneys at P125 revealed major sex-specific gene expression differences between male and female kidneys independent of *Pkd2* status. When late *Pkd2* deletion was superimposed on these sex-related alterations in gene expression, the transcriptional response was similar, albeit reduced, in females, suggesting shared cystogenic pathways in both sexes. In males with more advanced cystic disease, the most highly enriched biological processes were associated with metabolism (including fatty acid and carboxylic acid metabolism), chemotaxis, and cell migration, which is similar to what has been reported in other mouse models.^20,21,48-50^ Although our study was not designed to address this, we speculate that gene expression analysis of female mice at a later stage would have yielded similar results. Further emphasizing the importance of sex, we found that the transcription factor ESR1 was highly correlated with DEGs in both sexes. In addition, ESR1 protein levels were increased in *Pkd2* mutant male and female kidneys compared with controls. ESR1 regulates estrogen-responsive genes involved in sexual development, energy homeostasis, and glucose metabolism in both sexes.^51^ Interestingly, previous studies using non-orthologous PKD models observed that androgens were a disease progression factor, whereas estrogens had a protective role.^52,53^ It is possible that the estrogen-mediated signaling may act as a brake on cystogenic pathways, particularly in female mice.

In this report, we sought to define a transcriptional signature that correlates with PKD severity. Not unexpectedly, we found that DEGs related to proliferation pathways were enriched in kidneys with the largest KBW ratios, whereas pathways related to the immune response had little to no correlation with KBW ratios. These findings are consistent with a model in which inflammatory processes play a role in the initial steps of cystogenesis, whereas cell proliferation is associated with disease progression.

We conducted a meta-analysis of our data with 2 publicly available RNA-seq datasets selected using stringent criteria to identify core genes associated with *Pkd2* loss.^39,41^ These datasets were derived from a *Pkd2* mutant cell line and a *Pkd2* conditional model similar to ours, but at an earlier cystic stage. After reanalyzing the data to ensure consistency, we found that the transcriptional differences were driven more by the tissue of origin rather than genotype. The *Pkd2* mutant cell line exhibited the largest number of unique DEGs, and only 86 genes were differentially expressed across all 3 datasets. The 2 kidney datasets, which segregated more closely in 1 principal component analysis, shared 439 DEGs, 383 of which overlapped in the joint analysis of all the datasets (“joint all”). These genes were enriched for cell division and proliferation pathways and consensus sequences related to transcription factors Trp63 and NANOG. Trp63 regulates renal epithelial morphogenesis postinjury, whereas NANOG maintains pluripotency by activating repressors and suppressing activators of cell differentiation in embryonic stem cells and various cancers, including kidney.^54-60^ The role of NANOG and Trp63 in cystic progression deserves further investigation.

Our analyses also highlight the limitations of combining data across studies. Significant differences detected in a meta-analysis may reflect dataset-specific features to 1 of the datasets rather than a general feature of the disease. Likewise, when combining data from sources that vary in tissue type, genotype, sex, model, or degree of PKD progression, it is important to consider whether the meta-analysis is uncovering common pathways or potentially obscuring true differences. Ultimately, deciding which is true will require experimental validation. On the basis of our meta-analysis, cell-based models lacking the complexity of whole organisms may not fully recapitulate the molecular programs observed in cystic kidney, thereby limiting their utility for predicting therapeutic responses.

In conclusion, our results show that the kinetics of cyst formation in a *Pkd2* conditional mouse model are nearly identical to what has been described for *Pkd1* and that the models have similar transcriptional profiles. *Pkd2* conditional mice can therefore serve as a complementary model to *Pkd1* for understanding ADPKD pathogenesis and for testing therapeutics. Additionally, we show that an inducible system, such as the Cre *Pax8-rtTA; TetO-Cre,* which allows for temporal control of *Pkd* gene inactivation, permits modeling of adult-onset disease. Our findings confirm that sex is an important modifier of ADPKD progression in the kidney and implicate estrogen-related pathways in this process. Our data highlight the importance of sex as a biological variable that should be considered in any study design using orthologous mouse models with late *Pkd2* inactivation.

## Supplementary Material

MMC1

Supplementary material is available online at www.kidney-international.org.

## Data Availability

The RNA-sequencing datasets generated for this study can be found in the Gene Expression Omnibus archive (https://www.ncbi.nlm.nih.gov/gds/) with accession number GSE261420. Access to the data was restricted while the manuscript was reviewed; however, data will be made available to reviewers on request to the Editor. Supplementary Table S1 and Supplementary Table S2 can be accessed via the following link: https://figshare.com/s/20c86e78a7d961547875.

## Translational Statement

Mouse models are indispensable tools for studying autosomal dominant polycystic kidney disease (ADPKD), which is caused by mutations in *PKD1* or *PKD2*. We assayed the kinetics of cyst formation in a *Pkd2* mouse model that allowed us to control the timing of *Pkd2* inactivation. We found that similar to *Pkd1*, inactivation of *Pkd2* after postnatal day 14 resulted in delayed cyst formation that was more severe in males versus females. ADPKD progression in this model was associated with alterations in genes involved in metabolism, cell proliferation, and the immune response. There were remarkable differences in gene expression between male and female kidneys that were independent of the *Pkd2* genotype. Taken together, these findings suggest that *Pkd2* conditional mice are robust models for exploring ADPKD treatment and pathogenesis. Our results are important because they inform the design of preclinical studies using *Pkd2* mouse models.
