# Supplementary material for: Inactivation of Pkd2 in adult mice results in delayed cyst formation and identifies sex as a major modifier of disease severity
Source: Kidney Int. Author manuscript; Available in PMC 2026 Aug 7. (PMC13449052; doi:10.1016/j.kint.2025.08.039)
Supplement: Supplementary file 1 [file NIHMS2184642-supplement-MMC1.docx]

**Supplementary Figures**

**Supplementary Methods**

**Inactivation of Pkd2 in adult mice results in delayed cyst formation and identifies sex as a major modifier of disease severity.**

***Patricia Outeda^1*^, Perry Summers^1^, Denis Basquin^1^, Ruizhen Xu^1^, Jessica Hunt^1^, Luis F. Menezes^2^* and Terry Watnick^1*^***

**Supplementary Figures**

**Figure 1 (a)** **Schematic representation of kidney samples analyzed in Figure 1a-d. Colored dots indicate the collection time points**: Red dot: kidneys from mice induced at P10 and harvested at P20, N=8; Green dot: kidneys from mice induced at P11 and harvested at P21, N=6; Purple dot: kidneys from mice induced at P12 and harvested at P22, N=9; orange dot: kidneys from mice induced at P13 and harvested at P23, N=11; Blue dot: kidneys from mice induced at P14 and harvested at P24, N=11; Pink dot: kidneys from mice induced at P15 and harvested at P25, N=10. Scale bar, 2 mm. Only 1 kidney per mouse was used to determine KBW ratios and cystic index. (**b**) PCR analysis and (**c**) quantification showing the degree of *Pkd2* deletion in P13 (red dots, N=7) and P18 (pink dots, N=8) kidney tubules isolated from mice induced with Doxycycline at P10 and P15, respectively. *Pkd2* deletion efficiency was determined by calculating the ratio (percentage) of the densitometric intensity of the 209 bp floxed-deleted band to the total densitometric intensity of the flox-undeleted 318 bp band plus the 209 bp deleted band. Statistical significance was determined using unpaired two-tailed t-test. Data is presented as mean ± SEM, no-significant, ns.

**Supplementary Figure S2. (a) Kidney Sections Analyzed in Figure 2a-d. Colored dots indicate the collection time points:** Blue square: kidneys from male mice induced at P15 and harvested at P60, N=14; Red circle: kidneys harvested from female mice induced at P15 and harvested at P60, N=6. (**b**) Kidneys (N=3) harvested from female mice induced at P15 and euthanized at P116 (green dot). Of the remaining three females included in the survival analysis, one died spontaneously at P116 and two others at P127. These kidneys could not be harvested due to tissue decomposition. Scale bar, 2 mm. Only 1 kidney per mouse was used to determine KBW ratios and cystic index.

**Supplementary Figure S3. Kidney Weight-To-Body Weight Ratio in Early Onset Models by Gender**. Quantitation of one kidney-to-body-weight ratio (KBW) (**a**) and cystic index (**b**) for males and females. Squares represent each individual male and circles represent individual females in each group. Colors represent the timing of inactivation with Doxycycline: Red-P10, Green-P11, Purple-P12, Orange-P13, Blue-P14 and Pink-P15. Statistical significance was determined using multiple group comparisons by ordinary one-way ANOVA followed by Tukey's multiple comparisons test. Data is presented as mean ± SEM, no-significant, ns.

**Supplementary Figure S4**. **Cre Recombinase Activity in** ***Pkd2^fl/fl^; Pax8^rtTA^; TetO^Cre^*; *mT/mG* Male and Female Mice Treated with Doxycycline at P15 and Harvested at P20**. (**a**) Representative sections of kidney cortex (top panel) and renal medulla (bottom panel) from males (N=4) and females (N=4) showing red fluorescence labeling (tdTomato, *mT*) in red and green fluorescence (eGFP, *mG*) in green. Sections were counterstained with DAPI (blue). GFP expression indicates Cre recombinase activity. We observed a similar pattern of Cre activity in the renal cortex and renal medulla in kidneys from males and females. Scale bar 75 μm.­ (**b** and **c**) *Pkd2* deletion efficiency in male and female mice. Western blot analysis (b) of P25 kidney lysates from male (top panel, N=7) and female (bottom panel, N=5) *Pkd2^fl/fl^; Pax8^rtTA^; TetO^Cre^* mice induced with Doxycycline at P15. Control mice treated with Doxycycline at P15 and harvested at P25 are included (N=3). The same controls were used in both blots, but were loaded separately. β-actin was used as a loading control. Quantification of PC2 expression for males (blue dots), females (red dots), and controls (green dots) is shown in panel (c). Statistical significance was determined using multiple group comparisons by ordinary one-way ANOVA, followed by Tukey's multiple comparisons test. Data is presented as mean ± SEM, no-significant, ns, *****P* ≤ 0.0001. *Note: Bottom panel: Uncropped Western blots corresponding to panel (b). *Note: PC2 dimers detected at a higher molecular weight (~200 kDa).

**Supplementary Figure S5. Fibrosis is not the Initial Driver of Cyst Formation in Pkd2 Deficient Kidneys**. (**a**) Representative Masson’s Trichrome–stained kidney sections from *Pkd2^fl/fl^; Pax8^rtTA^; TetO^Cre^* positive males (blue-square) and females (red circle) littermates induced with Doxycycline at P15 and harvested at P60. Scale bars, 2 mm (black, left) and 200 μm (yellow, right) **b**) Kidney sections from males (blue-square, N=4) and females (red circle, N=4) littermates induced at P15 and harvested at P60 stained with α-SMA antibody (green) and counterstained with DAPI (blue). The dotted white squares indicate the areas at higher magnification in the right panels. Scale bar 100 μm (20 μm, input) (**c**) Representative western blot of α-SMA and loading control vinculin from 4 males and 4 females. * Note uncropped blot for reference (**d**) Quantification of the relative expression of α-SMA to vinculin. Statistical significance was determined using unpaired two-tailed t-test. Data is presented as mean ± SEM, ***P* ≤ 0.01.

**Supplementary Figure S6.** **Cre Recombinase Activity in *Pkd2^fl/fl^; Pax8^rtTA^; TetO^Cre^*; *mT/mG* Mice Treated with Doxycycline at P10 and P15.** Representative sections of kidney cortex and renal medulla from (**a**) mice induced at P10 and tissue collected at P15 (N=4), showing red fluorescence labeling (tdTomato, *mT*) in red and green fluorescence (eGFP, *mG*) in green (middle panel) and merged *mT/mG* labeling counterstained with DAPI (in blue, bottom panel). GFP expression indicates Cre recombinase activity. Scale bar 75 μm.­

**Supplementary Figure S7. Ki67 staining in *Pkd2^fl/fl^; Pax8^rtTA^; TetO^Cre^* Mice Induced at P15 and Harvested at P60**. (**a**) Representative P60 kidney sections from male (left panels) and female (right panels) mice induced at P15 and stained with Ki67 to assess proliferation. The upper panels show co-staining with anti-Ki67 (cyan) and the distal tubule marker DBA (red); the lower panels show anti-Ki67 (magenta) and the proximal tubule marker LTL (green). Scale bars 150 μm. (**b**) Quantification of proliferating renal epithelial cells in distal tubules (Ki67^+^ DBA^+^) and proximal tubules (Ki67^+^ LTL^+^) from *Pkd2^fl/fl^; Pax8^rtTA^; TetO^Cre^* male (blue squares, N=13) and female (red dots, N=6) mice induced at P15 and harvested at P60. Statistical significance was determined using unpaired two-tailed t-test. Data is presented as mean ± SEM, ***P* ≤ 0.01, no-significant, ns, **P* ≤ 0.05.

**Supplementary Figure S8. (a) Kidney Sections Analyzed in Figure 4a-c and Used to Perform RNA Sequencing Studies.** Top panels: kidneys from male mice induced at P27 and harvested at P125, red square (mutant males, N=5), blue square (control males, N=5). Bottom panels kidneys from female mice induced at P27 and harvested at P125, red circle (mutant females, N=5), blue circle (control females, N=5). Scale bar, 2 mm. Only 1 kidney per mouse was used to determine KBW ratios and cystic index. (**b**) Kidney Sections Analyzed in Supplementary Figure S9a-d. Top panels: kidneys from male mice induced at P27 and harvested at P85, red square (mutant males, N=5), blue square (control males, N=5). Bottom panels kidneys from female mice induced at P27 and harvested at P85, red circle (mutant females, N=5), blue circle (control females, N=5). Scale bar, 2 mm. Only 1 kidney per mouse was used to determine KBW ratios and cystic index.

**Supplementary Figure S9. Sexual Dimorphism in P85 Cystic Kidney Disease Severity Following *Pkd2* Inactivation at P27**. Mice were administered Doxycycline at postnatal day 27 (P27) to induce Cre-mediated recombination, and kidneys were harvested at P85. (**a**) Representative H&E-stained kidney sections from *Pkd2^fl/fl^; Pax8^rtTA^; TetO^Cre^* show focal cystic lesions distributed throughout the renal parenchyma in males (red squares), whereas cyst burden in females (red dots) is minimal or undetectable. (**b**) Kidney-to-body weight (KBW) ratio, (**c**) cystic index, and (d) blood urea nitrogen (BUN) levels from mutant male (red squares, N=5) and control littermates (blue squares, N=5) and mutant female (red circles, N=5) and control (blue circles, N=5) littermates. Statistical significance was determined using multiple group comparisons by ordinary one-way ANOVA followed by Tukey's multiple comparisons test. Data is presented as mean ± SEM, no-significant, ns, **P* ≤ 0.05, *****P* ≤ 0.0001.

**Supplementary Figure S10. Sex-Specific Transcriptional Differences in Males and Females Enriched for Genes Associated with Estrogen Receptor 1 (ESR1)**. (**a**) Venn diagram showing the number of differentially expressed genes (adjusted *P*-value < 0.01) comparing males to females in either mutant (red) or control (blue) samples. (**b**) Plot showing the significance of transcription factors associated with the genes differentially expressed between males and females in either the control (blue) or mutant (red) groups. (**c**) Plot showing the significance of transcription factors associated with the genes differentially expressed between mutants and controls only in males. (**d**) Representative western blot showing ESR1_66_ and loading control in the kidney samples harvested at P125 used to perform RNA-seq experiments. Vinculin was used as a loading control. (**e**) Quantification of the relative expression level of ESR1_66_ to vinculin in females (left panel) and males (right panel) separated by genotype (mutant, red; control, blue). Statistical significance was determined using unpaired two-tailed t-test. Data is presented as mean ± SEM, no-significant, ns, **P* ≤ 0.05, ****P* ≤ 0.001 Uncropped blots are included for reference.

**Supplementary Figure S11.** **Meta-analysis of the *Pkd2* Transcriptome.** (**a**) Venn diagram showing the number of differentially expressed genes (adjusted *P*-value < 0.01) comparing datasets used in the meta-analysis. (**b**) Plot showing LogFoldChange of gene expression differences between mutants and controls in Somlo (x-axis) versus Outeda (y-axis) datasets. Genes with log fold change > 5 or with log fold change difference between datasets above 3 (off-diagonal) are labeled.

**Supplementary Figure S12. Transcription Factor Enrichment in Kidney Data Sets.**

(**a**) Volcano plot showing the fold-change and adjusted p-value of the 22 NANOG (orange) and 8 TRP63 (blue) predicted targets among the 383 genes that were differentially expressed in both kidney datasets and in the joint analysis of all datasets (“joint all”). Each panel shows log2(fold-change) and -log10(adjusted p-value) obtained with the analysis within the corresponding dataset. (**b**) Network analysis showing genes associated with NANOG and their corresponding functional classification.

**Supplementary Figure S13. Network Analyses Showing Protein-Protein Interactions in the Kidney Datasets and Joint Analysis.** (**a**) Bar plot showing the size of clusters of interacting proteins identified in an analysis of the STRING protein-protein interaction database using the 383 genes differentially expressed in the two kidney datasets and in the joint analysis (“joint all”). (**b**) Volcano plot showing log2(fold-change) of mutant to control in the joint analysis (x-axis) and corresponding -log10(adjusted-p-value) (y-axis) of genes with at least one interaction in the STRING database. Dot size represents the number of protein-protein interactions. Dots in blue are part of the 21-protein sub-network and in orange of the 9-protein sub-network.

**Supplementary Figure S14. Network Analyses Showing Protein-Protein Interactions for Kidney Datasets**. (**a**) Sub-network of 21 proteins and corresponding pathway enrichment (**b**), showing the ratio of genes in the network to genes in the category (x-axis) and colored based on the adjusted p-value of pathway enrichment. (**c**) Sub-network of 9 proteins and corresponding pathway enrichment dot plot (**d**).

Link to Supplementary Table S1 and Supplementary Table S2 <https://figshare.com/s/20c86e78a7d961547875>

**Supplementary Methods**

**Animal Care and Use**

The *Pkd2* conditional allele (*Pkd2^fl/fl^*) used in these studies has been previously described [1]. The *Pkd2^fl/fl^; Pax8^rtTA^; TetO^Cre^* mouse model is an orthologous mouse model of ADPKD that uses the murine *Pax8* promoter to direct expression of the reverse tetracycline-dependent transactivator (rtTA) to all renal tubular compartments (proximal and distal tubules as well as collecting ducts) [2]. To induce *Pkd2* deletion, experimental mice were treated with a single daily intraperitoneal (IP) injection of Doxycycline Hyclate (Sigma-Aldrich, D9891) 50 mg/Kg body weight, diluted in sterile distilled water. Specific time points for induction are indicated in the figures. Littermate controls lacked either the *Pax8^rtTA^* or the *TetO^Cre^* module but were treated with Doxycycline in the same manner as experimental mice. To assess the activity of the Cre recombinase, we introduced the *mT/mG* reporter allele into the *Pkd2^fl/fl^; Pax8^rtTA^; TetO^Cre^* background by breeding (resulting in *Pkd2^fl/fl^; Pax8^rtTA^; TetO^Cre^*; *mT/mG* mice). The *mT/mG* reporter ubiquitously expresses the membrane-bound tdTomato (tandem dimer Tomato, *mT*), which emits red fluorescence. Upon Cre-mediated recombination, the *mT* cassette is excised and replaced by the membrane-targeted green fluorescent protein (*mG*), resulting in green fluorescence [3]**.** Mice included in the RNA-seq analysis were treated for 3 consecutive days (from P27 to P29) with a daily IP dose of Doxycycline Hyclate (50mg/Kg body weight) diluted in sterile distilled water. All mice were maintained on an inbred C57BL/6 background. Mice of both sexes were included in this study.

**Genotyping analysis**

Genomic DNA was isolated from mouse tails/toes and genotyped using the following primers: Pkd2flox11-13(A)-F 5’-CCTTTCCTCTGTGTTCTGGGGAG-3’, and Pkd2flox11-13(B)-R 5’-GTTTGATGCTTAGCAGATGATGGC-3’ to identify the *Pkd2^f/f^* allele; oIMR7385 5’-CCATGTCTAGACTGGACAAGA-3’ and oIMR7386 5’-CTCCAGGCCACATATGATTAG-3’ to identify the presence of the Pax8^rtTA^ transgene and Cre-200F: 5’- AGGTTCGTTCACTCATGGA-3’ and Cre-200R: 5’- TCGACCAG TTTAGTTACCC-3’ to identify Cre positive and negative mice. The PCR conditions used were 56°C, 63°C, and 59°C annealing temperature and 35 PCR cycles for *Pkd2^fl/fl^* and *Pax8^rtTA^* and *TetO^Cre^* transgenes respectively. The PCR products were resolved on 1.5% NuSieve agarose gels, yielding a product of ~318 base pairs, ~595 base pairs, and ~235 base pairs for *Pkd2^fl/fl^* and *Pax8^rtTA^* and *TetO^Cre^* transgenes respectively.

**Pkd2 deletion efficiency in renal tubules**

The efficiency of Pkd2 deletion was assessed using tubules isolated from *Pkd2^fl/fl^; Pax8^rtTA^; TetO^Cre^* mice induced at P10 and P15 and harvested prior to cyst formation at P13 and P18, respectively.

Tubule isolation was performed as described previously [4]. Briefly, pups were euthanized, and their kidneys were dissected, transferred to a cold PBS-filled dish, decapsulated, and sectioned into 4–5 thin transversal slices. Kidney slices were immersed in 1 mL of pre-warmed incubation buffer (2 mg collagenase type II dissolved in F12/DMEM medium) in a 2 mL Eppendorf tube and shaken in a Thermoblock at 37°C and 850 rpm for 7 minutes. The first supernatant (~800 µL), primarily containing debris, was discarded. A fresh 1 mL aliquot of pre-warmed incubation buffer was added to the remaining pellet, and the samples were incubated again under identical conditions. The supernatant from this second incubation was collected and transferred to a clean Eppendorf tube placed on ice for 5 minutes, allowing the tubules to settle. Meanwhile, the kidney slices in the original Eppendorf tube were incubated a third time with an additional 1 mL of pre-warmed incubation buffer under the same conditions. After 5 minutes, the supernatant from the ice-cooled tube was carefully removed, and 1 mL of cold PBS was added to the pellet. Following another 5-minute settling period, the supernatant was discarded. This washing step was repeated 2–3 more times to maximize tubule yield.

To evaluate Pkd2 deletion efficiency, we adapted a previously published protocol for assessing *Pkd1* deletion using the same Cre mouse strain [5]. DNA was extracted from isolated tubules using DNeasy Blood & Tissue Kit (Qiagen, Cat. no. 69504). The concentration of extracted DNA was measured with a Nanodrop 2000 spectrophotometer (Thermo Fisher Scientific, Cat. no. ND2000), and 50 ng/µL aliquots were prepared for each sample. PCR was performed using primers Pkd2flox11-13(A)-F (5’-CCT TTC CTC TGT GTT CTG GGG AG-3’), Pkd2flox11-13(B)-R (5’-GTT TGA TGC TTA GCA GAT GAT GGC-3’), and Pkd2flox11-13(D)-R (5’-CTG ACA GGC ACC TAC AGA ACA GTG-3’). The undeleted *Pkd2^f/f^* allele produced a 318 bp PCR product, while the recombined allele yielded a 209 bp product. PCR products were resolved on a 1.5% NuSieve agarose gel and visualized using a ChemiDoc XRS imaging system. The intensity of the PCR bands was quantified using ImageJ software (NIH). The deletion efficiency was calculated by determining the ratio (as a percentage) of the densitometric intensity of the floxed-deleted 209 bp band to the total densitometric intensity of the flox-undeleted 318 bp plus the floxed-deleted 209 bp band.

**Histology-Immunohistochemistry**

Mice were euthanized and kidneys were fixed with 4% formaldehyde methanol-free (Pierce™, #

28908) overnight and embedded in paraffin, sectioned, and stained with hematoxylin-eosin (H&E) using standard protocols. Images were photographed by a digital camera (Infinity 2) connected to a Nikon eclipse E600 upright microscope. Cystic Index calculation: Hematoxylin and eosin (H&E)-stained sections of whole kidneys, sectioned transversely, were imaged using a 4X objective. Images were then converted to 8-bit grayscale, and the thresholding function in ImageJ (Fiji, Madison, WI) was used to differentiate cystic lumens from the surrounding tissue. The cystic index was calculated by measuring the total area occupied by cystic lumens and dividing it by the total kidney area (including both cystic and non-cystic regions). This value was then expressed as a percentage, providing a quantitative assessment of cyst burden for each sample. Cyst Origin Assessment: was performed by staining paraffin kidney sections using rhodamine-labeled Dolichos Biflorus Agglutinin (DBA) and fluorescein-labeled Lotus Tetragonolobus Lectin (LTL) (Vector Laboratories, #RL-1032 and #FL-1321 respectively). Nuclei were counterstained with DAPI, washed three times with PBST, and mounted with Fluoromount-G™ Mounting Medium (Invitrogen, #00-4958-02). Quantification of Proliferation of DBA and LTL cells: in specific tubular nephron segments was performed by staining paraffin-embedded kidney sections with DBA and LTL markers as described above and proliferating cells were identified by immunostaining with an anti-Ki67 antibody (Abcam, #ab15580), a marker of actively cycling cells. Nuclei were counterstained with DAPI. Images were acquired using a W1 confocal microscope and proliferation was quantified by counting the number of Ki67-positive nuclei within DBA- or LTL-positive tubules. At least 6 fields per section were analyzed, and data were expressed as the percentage of Ki67-positive nuclei relative to the total number of DAPI-stained nuclei in each tubule segment**.** Interstitial fibrosis assessment: interstitial fibrosis was assessed using 5 μm paraffin sections and stained with Masson's trichrome using standard protocols. Fibrotic tissue was included as a positive control to validate staining standards. Myofibroblasts were identified as interstitial spindle-shaped cells stained with α-SMA antibody (Clone 1A4, Sigma #A2547; 1:200) and Alexa-Fluor 488-labeled donkey anti-mouse IgG (Invitrogen #A21202; 1:1000). Sections were counterstained with DAPI. Slides were washed with PBST and mounted with Fluoromount-G™ Mounting Medium. Tissue Preparation and histology to assess *mT/mG* expression: kidneys were fixed overnight in 4% PFA at 4°C, cryoprotected using a dilution series of 15%-25%-30% sucrose at 4°C, embedded in Tissue-Tek^®^ O.C.T. Compound (Sakura, # 4583) and stored at -80°C. Eight-micrometer sections were counterstained with DAPI, washed three times with PBS, and mounted with Fluoromount-G™ Mounting Medium.

All images were photographed using a Nikon W-1 spinning disk confocal microscope at the UMB-SOM Confocal Microscopy Core- Baltimore, Maryland. All histological analyses were performed by blinded observers.

**Blood urea nitrogen (BUN) measurements**

Serum BUN levels were measured using either a colorimetric assay kit (ThermoFisher Scientific, Cat# EIABUN) according to the manufacturer’s instructions or by Antech Diagnostics (Morrisville, NC) using an automated enzymatic assay on a Beckman Coulter clinical chemistry analyzer. All BUN measurements in a given figure were performed using the same assay.

**Western Blot analysis**

Frozen tissues were lysed using Cell Lysis Buffer [20 mM Na-P (pH 7.2), 150 mM NaCl, 1mM EDTA, 10% Glycerol, 1% Triton-X100] and a cocktail of protease inhibitors (Sigma, P8340, 1:100). The lysates were homogenized with a motor driven Polytron, incubated for 40 minutes at 4°C rotating in lysis buffer and centrifuged at 14k rpms for 30 minutes at 4°C. The supernatant was transferred to a fresh tube and the protein concentration was measured using the BCA protein assay kit (Pierce). Forty to fifty micrograms of protein were loaded on NuPAGE™ 3%-8%, Tris-Acetate mini protein gels (Invitrogen™, #EA03755BOX). Proteins were blotted using α-SMA antibody (Sigma, #A2547, Clone 1A4; 1:1000), Polycystin 2 antibody (3374 kindly provided by Dr. Qian, PKDRRC; 1:1000), β-Actin (13E5) (Cell Signaling, #93473, 1:5000) and vinculin antibody (Sigma, #V9131, clone hVIN-1; 1:2000) and secondary HRP-linked whole anti-mouse and anti-rabbit antibodies, (Amersham, NA-934, 1:10000). Blots were imaged using a GelDoc Go imaging system (Bio-Rad) and band density was quantified using ImageJ (Fiji, Madison, WI).

**RNA Isolation**

Kidneys from *Pkd2^fl/fl^; Pax8^rtTA^; TetO^Cre^* positive and negative controls from males and females (N=5 per group) induced from P27 to P29 as indicated above were collected and snap frozen at P125 for RNAseq-analysis. Total RNA was isolated using Trizol (Fisher Scientific) and Qiagen RNeasy kit (Quiagen, #74034) using the manufacturer’s protocols. RNA libraries were generated, and RNA sequencing was conducted by a core laboratory at the Institute for Genome Sciences (University of Maryland) using the NovaSeq S4 flow cell (Illumina).

**Meta-analyses and Datasets**

The datasets used for the meta-analysis are available for download from the GEO database [6] with identifiers GSE149739 [7] and GSE171574 [8]. Other datasets with identifiers GSE151951, GSE134721, and GSE86509 were used for partial analysis but were excluded from the final analysis because they failed to meet inclusion criteria [9-11]: 1) RNA-seq data available for download with at least 3 *Pkd2* knockout and control samples; 2) *Pkd2* expression consistently lower than available controls; and 3) to avoid inflating data with false positives, less than 30% of genes differentially expressed between mutants and controls. Fastq sequence files were downloaded with sratoolkit 3.0.5 and pre-processing and analysis were done as described above. The datasets were analyzed separately (comparing genotypes within sex or cell passage within the samples in the original study) and also together (comparing genotypes within the study and group).

**Statistics**

Values were expressed as mean ± SEM. Statistical comparisons were done using the two-tailed unpaired *t*-test with Welch’s correction when two groups were compared, or one-way ANOVA followed by Tukey’s multiple comparisons test using GraphPad Prism 9.0 software when more than 2 groups were compared. The probability value of *P* ≤ 0.05 was considered significant.

**References**

1.	Garcia-Gonzalez, M.A., et al., *Pkd1 and Pkd2 are required for normal placental development.* PLoS One, 2010. **5**(9).

2.	Traykova-Brauch, M., et al., *An efficient and versatile system for acute and chronic modulation of renal tubular function in transgenic mice.* Nat Med, 2008. **14**(9): p. 979–84.

3.	Muzumdar, M.D., et al., *A global double-fluorescent Cre reporter mouse.* Genesis, 2007. **45**(9): p. 593–605.

4.	Belavgeni, A., F. Maremonti, and A. Linkermann, *Protocol for isolating murine kidney tubules and ex vivo cell death assays.* STAR Protoc, 2024. **5**(2): p. 103005.

5.	Zhang, C., et al., *Glis2 is an early effector of polycystin signaling and a target for therapy in polycystic kidney disease.* Nature Communications, 2024. **15**(1): p. 3698.

6.	Clough, E., et al., *NCBI GEO: archive for gene expression and epigenomics data sets: 23-year update.* Nucleic Acids Res, 2024. **52**(D1): p. D138–d144.

7.	Zhang, C., et al., *Cyclin-Dependent Kinase 1 Activity Is a Driver of Cyst Growth in Polycystic Kidney Disease.* J Am Soc Nephrol, 2021. **32**(1): p. 41–51.

8.	Jung, H.J., et al., *Polycystin-2-dependent transcriptome reveals early response of autosomal dominant polycystic kidney disease.* Physiol Genomics, 2023. **55**(11): p. 565–577.

9.	Dixon, E.E., et al., *GDNF drives rapid tubule morphogenesis in a novel 3D in vitro model for ADPKD.* J Cell Sci, 2020. **133**(14).

10.	Lee, E.C., et al., *Discovery and preclinical evaluation of anti-miR-17 oligonucleotide RGLS4326 for the treatment of polycystic kidney disease.* Nat Commun, 2019. **10**(1): p. 4148.

11.	Woo, Y.M., et al., *Profiling of miRNAs and target genes related to cystogenesis in ADPKD mouse models.* Sci Rep, 2017. **7**(1): p. 14151.
